# Interleukin 37 promotes angiogenesis through TGF-β signaling

**DOI:** 10.1038/s41598-017-06124-z

**Published:** 2017-07-21

**Authors:** Mengmeng Zhao, Yongguang Hu, Jiayi Jin, Ying Yu, Shanshan Zhang, Jingjing Cao, Yuanfen Zhai, Rongbin Wei, Juanjuan Shou, Wenping Cai, Shangfeng Liu, Xiaoping Yang, Guo-Tong Xu, Jianhua Yang, David B. Corry, Shao Bo Su, Xialin Liu, Tianshu Yang

**Affiliations:** 1 Shanghai Tenth People’s Hospital, Tongji University School of Medicine, Shanghai, China; 2 State Key Laboratory of Ophthalmology, Zhongshan Ophthalmic Center, Sun Yat-sen University, Guangzhou, China; 3 Johns Hopkins University School of Medicine, Baltimore, United States; 4 Department of Pathology and Immunology, Baylor College of Medicine, Houston, United States

## Abstract

IL-37 is a novel pro-angiogenic cytokine that potently promotes endothelial cell activation and pathological angiogenesis in our previous study, but the mechanisms behind the pro-angiogenic effect of IL-37 are less well understood. Extending our observations, we found that TGF-β interacts with IL-37, and potently enhances the binding affinity of IL-37 to the ALK1 receptor complex, thus allowing IL-37 to signal through ALK1 to activate pro-angiogenic responses. We further show that TGF-β and ALK1 are required in IL-37 induced pro-angiogenic response in ECs and in the mouse model of Matrigel plug and oxygen-induced retinopathy. The result suggests that IL-37 induces pro-angiogenic responses through TGF-β, which may act as the bridging molecule that mediates IL-37 binding to the TGF-β receptor complex.

## Introduction

Angiogenesis, the sprouting of new vessel branches from the pre-existing vasculature, is tightly controlled by coordination of pro- and anti-angiogenic cytokines^1^. The multifunctional cytokine, transforming growth factor-β (TGF-β), has been recognized as a pivotal regulator during both developmental and patho-physiological angiogenesis^2^. The angiogenic activity of TGF-β is highly context-dependent and coordinated by many regulatory factors^3^. TGF-β binds to type II receptor (TβRII), which recruits type I receptors, termed activin receptor-like kinase (ALKs), to activate downstream signaling^4^. In vascular endothelial cells, the angiogenic activity of TGF-β elicits pro-angiogenic responses primarily through endothelial cell (EC)-restricted TGF-β type I receptor, ALK1, leading to phosphorylation of Smad1/5/8 and activation of pro-angiogenic signaling pathways^2, 5, 6^. Deletion of components of this pathway in murine models has been linked with abnormalities in blood vessel formation and stabilization, suggesting a critical role for ALK1 signaling in angiogenesis^6^. Knockout of ALK1 target genes results in reduced tumor growth and metastasis due to poor vascularization^7^. ALK1 inhibitors, which were demonstrated to suppress VEGF-induced EC proliferation and tumor growth, are currently being evaluated in clinical trials^8, 9^. However, the regulatory role of ALK1 in angiogenesis has proved to be highly context-dependent. Ectopic expression of a constitutively active form of ALK1 in ECs inhibits proliferation and migration^10^. Mice treated with ALK1-Fc trap or EC-specific ALK1 knockout mutants displayed excessive angiogenesis in developmental mouse retina, suggesting an anti-angiogenic role of ALK1^11, 12^. Possible reasons for these opposing results may rely on the involvement of multiple ligands and receptors.

IL-37, a recently identified anti-inflammatory cytokine of the IL-1 family, has been reported to be a potent suppressor of immune response^13, 14^. IL-37 expression is detected in lymph nodes, thymus and bone marrow, as well as in monocytes, epithelial cells, breast carcinoma cells and endothelial cells^15, 16^. As an inflammatory cytokine, IL-37 regulates immune responses both intracellularly and extracellularly. Intracellular overexpressed IL-37 was suggested to interact with Smad3 to exert its inflammatory function^13^. Extracellular IL-37 was suggested to bind to IL-18Rα and IL-1R8, which function together to mediate the anti-inflammatory activity of IL-37^17, 18^. IL-37 is reported to be involved in a variety of angiogenesis-associated diseases. IL-37 is detected in tumor cells of breast carcinoma and infiltrating plasma cells in colon carcinoma^19^. Moreover, IL-37 is upregulated in the synovial tissue and serum from patients with rheumatoid arthritis^13, 20^.

Our previous study showed that IL-37 function as a potent pro-angiogenic cytokine with compared potency to that of vascular endothelial growth factor (VEGF)^21^. IL-37 enhances EC proliferation, migration and capillary formation *in vitro*. In mice, IL-37 enhanced angiogenesis in Matrigel plug assay and promoted neovascularization in oxygen-induced retinopathy and in neonatal retinal vasculature. However, the molecular mechanism underlying the pro-angiogenic effect of IL-37 remains unknown. In this study, we find that IL-37 employs a novel mechanism to induce pro-angiogenic signaling. We provide evidence that IL-37 binds to TGF-β, which markedly enhances the binding of IL-37 to the ALK1 receptor complex and allows IL-37 to signal through ALK1 to activate pro-angiogenic responses. Blockade of TGF-β1 or ALK1 resulted in decreased responses to IL-37 *in vitro* and *in vivo*. These results unveiled a novel mechanism underlying IL-37-indueced pro-angiogenic responses.

## Results

### IL-37 is upregulated by TGF-β in HUVECs

**Figure 1:**
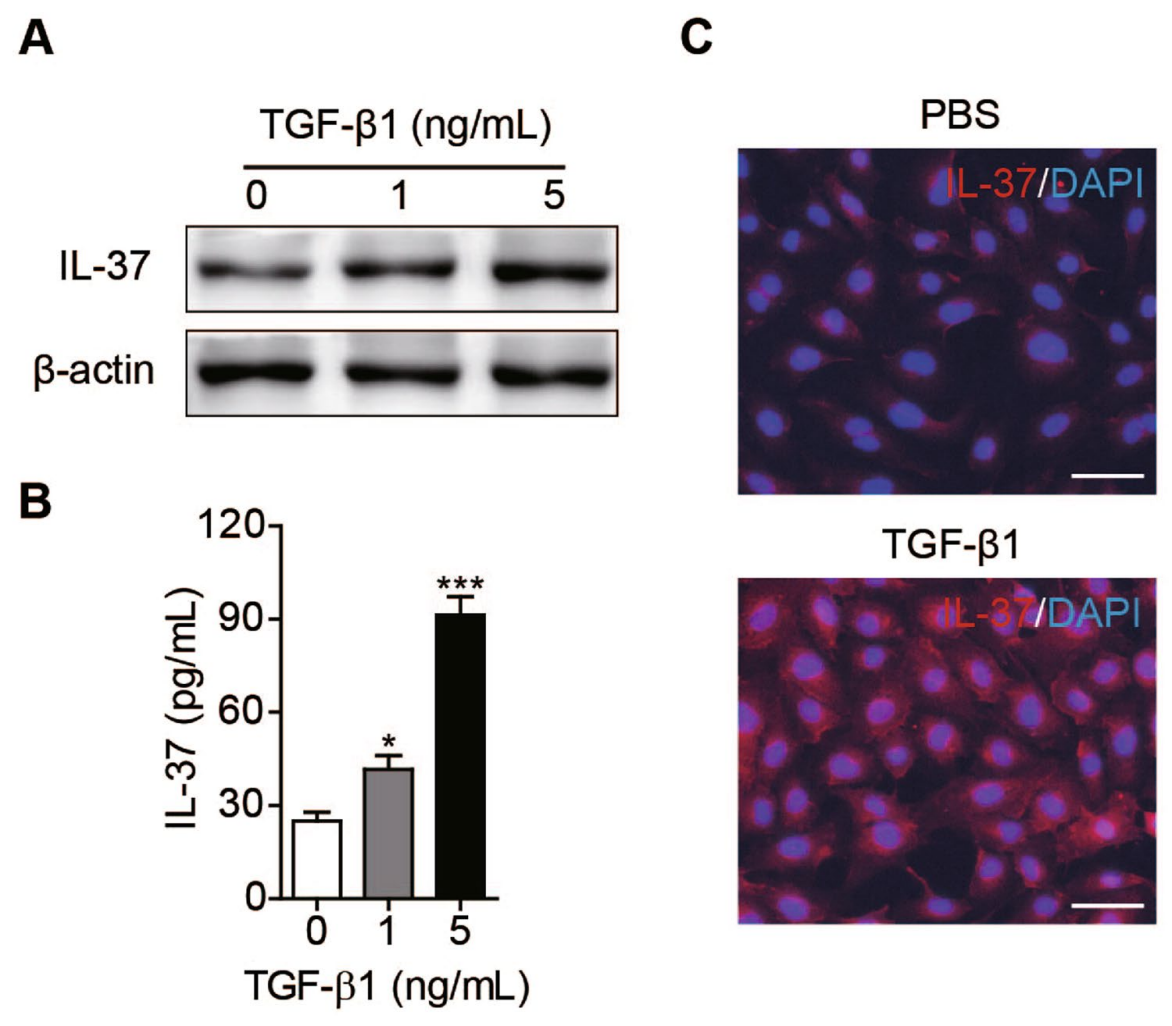
IL-37 expression in endothelial cells was upregulated by TGF-β. (**A**) HUVECs were pre-starved under serum-free conditions without supplemented growth factors overnight and then treated with indicated concentrations of TGF-β1 for 12 hours. IL-37 expression was examined by Western blot. Blots are representative of three experimental replicates. (**B**) Quantification of IL-37 in the supernatants of HUVECs when treated with indicated concentrations of TGF-β1 determined by ELISA. *n* = 4 per group. (**C**) Immunofluorescent staining of cultured HUVECs for IL-37 after treated with PBS or TGF-β1. Scale bar, 50 μm. Data are presented as mean ± SEM (*n* = 4 per group). *P < 0.05. **P < 0.01.

In our previous study, we showed that both IL-37 transcripts and protein expression were detected in large and small vascular endothelial cells including HUVECs and human microvascular endothelial (HMEC-1) cells^21^. Of note, previous studies showed that IL-37 expression was upregulated by TGF-β treatment in PBMCs and THP-1 cells^13^. Consistently, we observed that IL-37 expression and secretion in HUVECs were upregulated by TGF-β1 as determined by Western blot and ELISA (Fig. 1A,B). By immunofluorescence, IL-37 was barely detectable in HUVECs, but HUVECs stimulated by TGF-β1 showed increased expression of IL-37 (Fig. 1C). Additionally, the effect of other members in TGF-β family on IL-37 expression in HUVECs was determined. The result showed that TGF-β1 rather than BMP10 or GDF-11 upregulated IL-37 expression in HUVECs (Fig. S1). These results suggest that IL-37 is upregulated by TGF-β in endothelial cells.

### TGF-β enhances the binding of IL-37 to HUVECs

As IL-37 was suggested to bind to IL-18Rα^18^, to determine whether this receptor mediates the pro-angiogenic responses to IL-37 in HUVECs, we started by competitive receptor binding assay to determine whether the binding of IL-37 to HUVECs could be competed with excessive IL-18. IL-37 was biotinylated and the bioactivity of biotinylated IL-37 (biot-IL-37) to stimulate HUVECs proliferation was comparable to that of unlabeled IL-37 (Fig. S2). The binding of biot-IL-37 bound to HUVECs and this binding was abolished by the addition of excessive unlabeled IL-37 (Fig. S3). However, when excessive unlabeled IL-18 was added to bio-IL-37 labeled HUVECs, IL-18 did not compete the binding of IL-37 to HUVECs, suggesting that IL-37 does not utilize the receptor of IL-18 to exert its function in HUVECs (Fig. S3).

As TGF-β is a pivotal cytokine regulating angiogenesis, we thus explored the possibility that IL-37 signals through TGF-β receptor in HUVECs. If this is true, TGF-β was hypothesized to compete the binding of IL-37 to HUVECs in competitive receptor binding assay.

**Figure 2:**
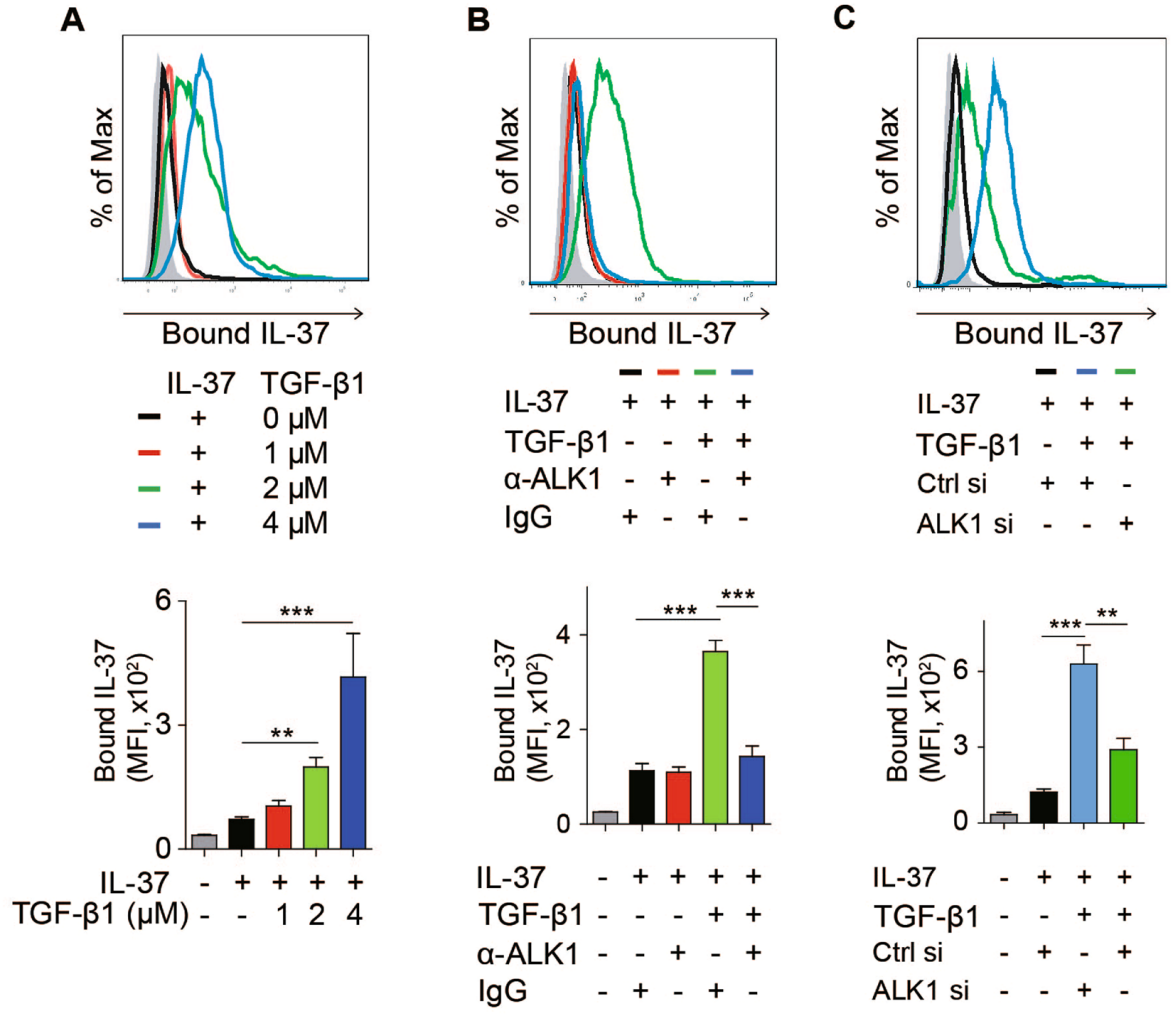
TGF-β1 enhanced the binding of IL-37 to HUVECs. (**A**) TGF-β1 facilitates the binding of IL-37 to HUVECs. HUVECs were incubated with 1 μM biot-IL-37 with/without indicated concentrations of TGF-β1. Bound biot-IL-37 was assessed by flow cytometry. Gray histograms indicated unlabeled cells. The mean fluorescence intensity (MFI) of bound IL-37 was quantified (*n* = 4). (**B**) ALK-1 mediates TGF-β-facilitated binding of IL-37 to HUVECs. Flow cytometry of HUVECs incubated with indicated cytokines or antibodies. Biot-IL-37 and TGF-β1 were used at 1 μM. Anti-ALK1 antibody (α-ALK1) and IgG were used at 10 μg/mL. (**C**) Control and ALK1 siRNA-transfected HUVECs were incubated with indicated proteins or antibodies (10 μg/mL). Biotinylated IL-37 (biot-IL-37) and recombinant TGF-β were used at 1 μM. Data are presented as mean ± SEM. **P < 0.01, ***P < 0.001.

Surprisingly, bound IL-37 to HUVECs was markedly increased when cells were incubated with increasing concentrations of TGF-β1 (Fig. 2A), indicating that TGF-β increased the binding affinity of IL-37 to HUVECs. In endothelial cells, TGF-β induces pro-angiogenic responses primarily through ALK1. Thus, to study the possibility that IL-37 may bind to HUVECs through the ALK1 receptor complex with the assistance of TGF-β, we blocked ALK1 with ALK1-neutralizing antibodies in receptor binding assays and found that TGF-β-facilitated binding of IL-37 was abolished (Fig. 2B), suggesting that ALK1 is required for the binding between IL-37 and HUVECs. Consistent with this observation, when ALK1 was knocked down with siRNA, TGF-β-facilitated binding of IL-37 to HUVECs was diminished as determined by flow cytometry, indicating the requirement of ALK1 in the binding of IL-37 to HUVECs (Fig. 2C). However, IL-37 did not facilitate the binding of TGF-β to HUVECs vice versa (Fig. S4), suggesting that IL-37 did not interfere the binding of TGF-β to its receptor. Together, these results suggest that TGF-β1 facilitates the binding of IL-37 to HUVECs.

### IL-37 interacts with TGF-β

**Figure 3:**
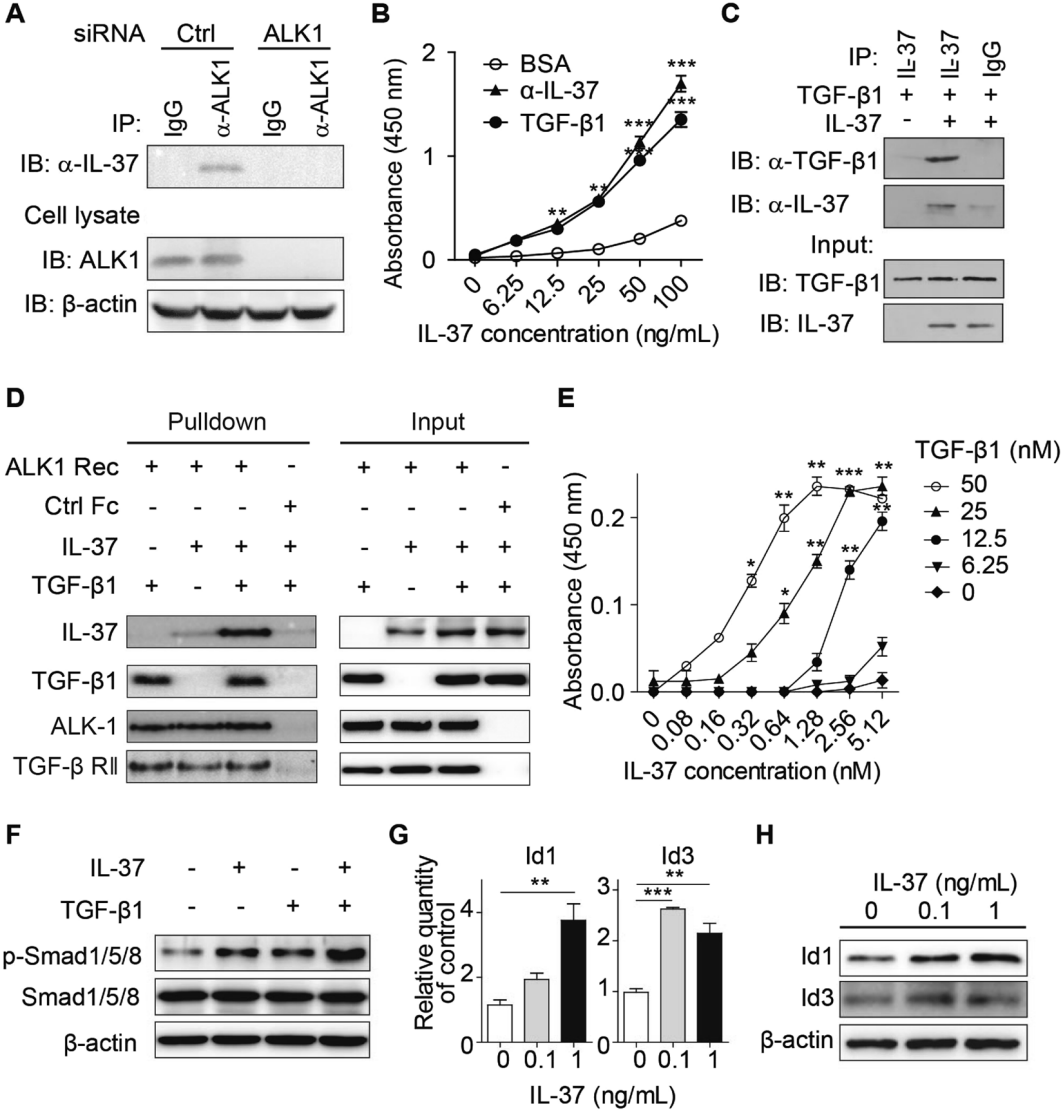
TGF-β1 enhanced the binding of IL-37 to the ALK1 receptor complex. (**A**) HUVECs were transfected with ALK1 siRNA or control (Ctrl) siRNA. Cell lysates were incubated with TGF-β and IL-37 and then subjected to co-immunoprecipitation with ALK1-specific antibodies. (**B**) Binding curves of IL-37 to immobilized TGF-β1. TGF-β1 (2 μg/mL), anti-IL-37 polyclonal antibodies (2 μg/mL) or BSA (2 μg/mL) were immobilized on 96-well plate and bound IL-37 was detected with antibodies. n = 4. (**C**) Interaction of IL-37 and TGF-β1 determined by *in vitro* pull-down assay. (**D**) TGF-β enhanced the binding between IL-37 and the ALK1 receptor complex (ALK1 Rec) in pull-down assay. The ALK1 receptor complex (pre-incubated ALK1-Fc and TβRII-Fc) or control Fc were conjugated to protein A/G beads, which were then incubated with IL-37 in the presence or absence of TGF-β1. (**E**) 96-well plates were coated with the ALK1 receptor complex, incubated with IL-37 and TGF-β, and bound IL-37 was detected by antibodies. (**F**) HUVECs were treated with IL-37 (1 ng/mL) in the presence or absence of TGF-β1 (5 ng/mL), and Smad1/5/8 phosphorylation was determined by Western blot. (**G** and **H**) IL-37 upregulated (**G**) mRNA levels and (**H**) protein levels of Id1 and Id3 in HUVECs. *n* = 4. Blots are representative of three experimental replicates. *P < 0.05, **P < 0.01, ***P < 0.001.

The result that TGF-β facilitates the binding of IL-37 to HUVECs through ALK1 suggests the possibility that IL-37 may utilize the receptor of TGF-β to exert its pro-angiogenic function. In endothelial cells, upon binding to TGF-β, the type II receptor (TβRII) recruits type I receptor ALK1, leading to formation of the ALK1 receptor complex and activation of pro-angiogenic signaling^4^. We thus performed co-immunoprecipitation (Co-IP) experiment to validate the interaction between IL-37 and ALK1 in HUVECs. HUVEC lysates were supplemented with IL-37 and TGF-β, and then subjected to immunoprecipitation. The result showed that ALK1 specific antibodies co-immunoprecipitated IL-37 in HUVEC lysates (Fig. 3A). When ALK1 was knocked down, the presence of IL-37 in ALK1-specific immunoprecipitate was markedly reduced. These observations suggest that IL-37 associates with the ALK1 receptor complex.

To answer the question whether IL-37 directly binds to the ALK1 receptor complex or indirectly through TGF-β, we first investigated whether IL-37 interact with TGF-β using immobilization binding assay. 96-well ELISA plates were coated with TGF-β1 and serial dilutions of IL-37 were added. Plate-bound IL-37 was determined using biotinylated IL-37 antibody. The result showed that IL-37 directly associates with TGF-β1, as compared to the positive control of anti-IL-37 capture antibodies, suggesting that IL-37 directly interacted with TGF-β1 (Fig. 3B). To further verify the interaction between IL-37 and TGF-β1, we performed *in vitro* pull-down assay and found that recombinant IL-37 was able to pull down TGF-β1, suggesting that IL-37 directly associated with TGF-β (Fig. 3C). These results showed that TGF-β interacts with IL-37, suggesting the possibility that TGF-β may bring IL-37 to the ALK1 receptor complex.

### TGF-β enhances the binding of IL-37 to the TGF-β receptor complex

To determine how TGF-β modulates the binding between IL-37 and the ALK1 receptor complex, we thus performed *in vitro* pull-down assay. Chimeric proteins of ALK1-Fc and TβRII-Fc were incubated to form the ALK1 receptor complex, which was then conjugated to protein A/G beads and incubated with IL-37 or/with TGF-β. Proteins associated with the ALK1 receptor complex were detected by Immunoblotting. The results showed that TGF-β potently enhanced the binding between IL-37 and the ALK1 receptor complex, suggesting that TGF-β brings IL-37 to the ALK1 receptor complex and is required for the association between IL-37 and the ALK1 receptor complex (Fig. 3D). Consistent with the result of receptor binding study (Fig. S4), IL-37 did not affect the binding of TGF-β to the ALK1 receptor complex (Fig. 3D). In endothelial cells, after binding to TβRII receptor, TGF-β stimulates either the pro-angiogenic ALK1 signaling or ALK5 signaling, which inhibits endothelial cell activation. To answer the question whether IL-37 could bind to ALK-5 receptor complex, we performed pull-down assay to determine the binding of IL-37 to ALK5. The results showed that the ALK5 receptor complex did not pulled down IL-37 either in the presence or in the absence of TGF-β (Fig. S5).

To further characterize the interaction between IL-37 and the ALK1 receptor complex, we performed kinetic immobilization binding assays to determine the effect of TGF-β on the binding between IL-37 and the ALK1 receptor complex. 96-well plates were coated with the ALK1 receptor complex, which is composed of the ectodomain of ALK1 and TβRII, and then incubated with increasing concentrations of IL-37 in the presence of different concentrations of TGF-β. As shown in Fig. 3E, the binding affinity between IL-37 and the ALK1 receptor complex was significantly increased by TGF-β. We then assessed the effect of different molar ratios of TGF-β to IL-37 on the binding of IL-37 to ALK1. As the molar ratio of TGF-β to IL-37 increased, TGF-β enhanced the binding affinity between IL-37 and the ALK1 receptor complex, reaching saturation at a molar ratio of 4:1 (Fig. S6A). In contrast, IL-37 did not affect the binding of TGF-β to the ALK1 receptor complex (Fig. S6B). Taken together, these data suggest that TGF-β increases the binding affinity between IL-37 and the ALK1 receptor complex and is required for the association between IL-37 and the ALK1 receptor complex.

### IL-37 activates Smad1/5/8 signaling

The results that TGF-β brings IL-37 to the ALK1 receptor complex suggest the possibility that IL-37 activates the pro-angiogenic signaling downstream of ALK1. When HUVECs were treated with combination of IL-37 and TGF-β1, Smad1/5/8 phosphorylation was increased compared to IL-37 or TGF-β1 treated cells, suggesting that IL-37 triggered downstream pro-angiogenic signaling of ALK1 synergistically with TGF-β (Figs 3F and S7). Consistent with the result of pull-down study (Fig. S5), in the presence of TGF-β1, IL-37 increased Smad1/5/8 phosphorylation but not Smad2/3 phosphorylation (Fig. S7). The results suggest that IL-37 preferably stimulates ALK1 activation in the presence of TGF-β1. Furthermore, IL-37 upregulated the expression levels of Id1 and Id3, the target genes of Smad1/5/8 as determined by real-time PCR and Western blot (Fig. 3G and H). These results indicate that IL-37 activated the pro-angiogenic ALK1-Smad1/5/8 signaling.

### IL-37 promotes angiogenesis through TGF-β signaling in ECs

**Figure 4:**
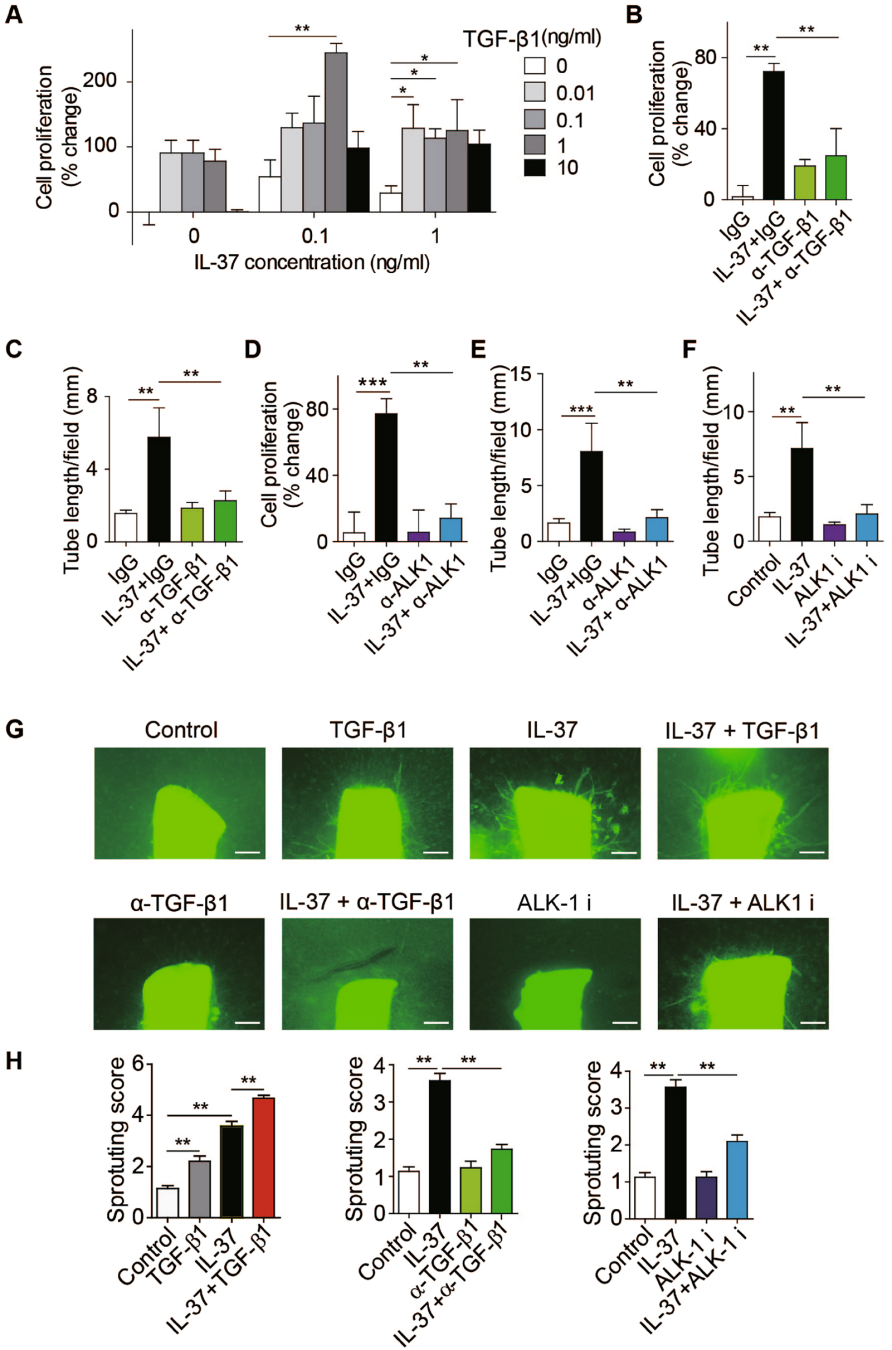
IL-37 promotes angiogenesis via TGF-β signaling pathway. (**A**) TGF-β1 augmented IL-37-induced cell proliferation in a dose-dependent manner. (**B** and **C**) TGF-β-neutralizing antibodies (α-TGF-β1, 10 μg/mL) suppressed IL-37 (1 ng/mL) induced (**B**) HUVEC proliferation (n = 5 per group) and (**C**) tube formation (n = 5 per group). (**D** and **E**) ALK1-neutralizing antibodies (α-ALK1) blocked IL-37 (1 ng/mL) induced (**D**) HUVEC proliferation (n = 6) and (**E**) tube formation (n = 6). (**F**) ALK1 inhibitor (ALK i) LDN193189 (0.5 μM) inhibited IL-37-stimulated Matrigel tube formation. n = 5 per group. (**G**) Blockade of TGF-β1 or ALK1 with neutralizing antibodies (10 μg/mL) or inhibitor (0.5 μM) inhibited IL-37 (1 ng/mL) stimulated vessel growth from aortic rings. n = 10 per group. (**H**) Aggregate analysis of the sprouting. n = 10 per group. Data are presented as mean ± SEM. *P < 0.05, **P < 0.01 and ***P < 0.001.

To address the synergistic effect of IL-37 and TGF-β, we have stimulated HUVECs with combinations of different concentrations of IL-37 and TGF-β1 in serum-free media. IL-37 or TGF-β alone induced limited increase in cell proliferation. Interestingly, the combined supplementation of IL-37 and TGF-β led to a remarkable increase in cell proliferation (Fig. 4A). When cells were treated with 0.1 ng/mL IL-37, TGF-β1 augmented IL-37-induced cell proliferation in a dose-dependent manner and the optimal dose of TGF-β1 was 1 ng/mL. Specific inhibition of TGF-β with neutralizing antibodies resulted in inhibited response to IL-37 in endothelial proliferation assay and tube formation assay (Figs 4B,C and S8), suggesting that the pro-angiogenic effect of IL-37 is critically dependent on TGF-β. These results suggest that IL-37 triggered pro-angiogenic signaling in synergy with TGF-β1.

As we showed that TGF-β potently enhanced the binding of IL-37 to the ALK1 receptor complex, we next sought to determine whether IL-37 promotes angiogenesis through TGF-β receptor. ALK1-neutralizing antibodies significantly blocked IL-37-induced proliferation and tube formation in HUVECs (Figs 4D,E and S8). Consistently, ALK1-specific inhibitor LDN193189 prevented IL-37-induced tube formation in HUVECs (Figs 4F and S8). To further confirm that the pro-angiogenic effect of IL-37 was mediated through the TGF-β-ALK1 pathway, either TGF-β or ALK1 was neutralized with antibodies, resulting in suppressed vessel growth in IL-37-treated aortic rings (Fig. 4G,H). Knockdown of ALK1 with siRNA also inhibited IL-37-induced sprouting in mouse aortic rings (Fig. S9). These results together suggest that IL-37 promotes pro-angiogenic responses through TGF-β signaling.

### IL-37 promotes angiogenesis through TGF-β signaling *in vivo*

**Figure 5:**
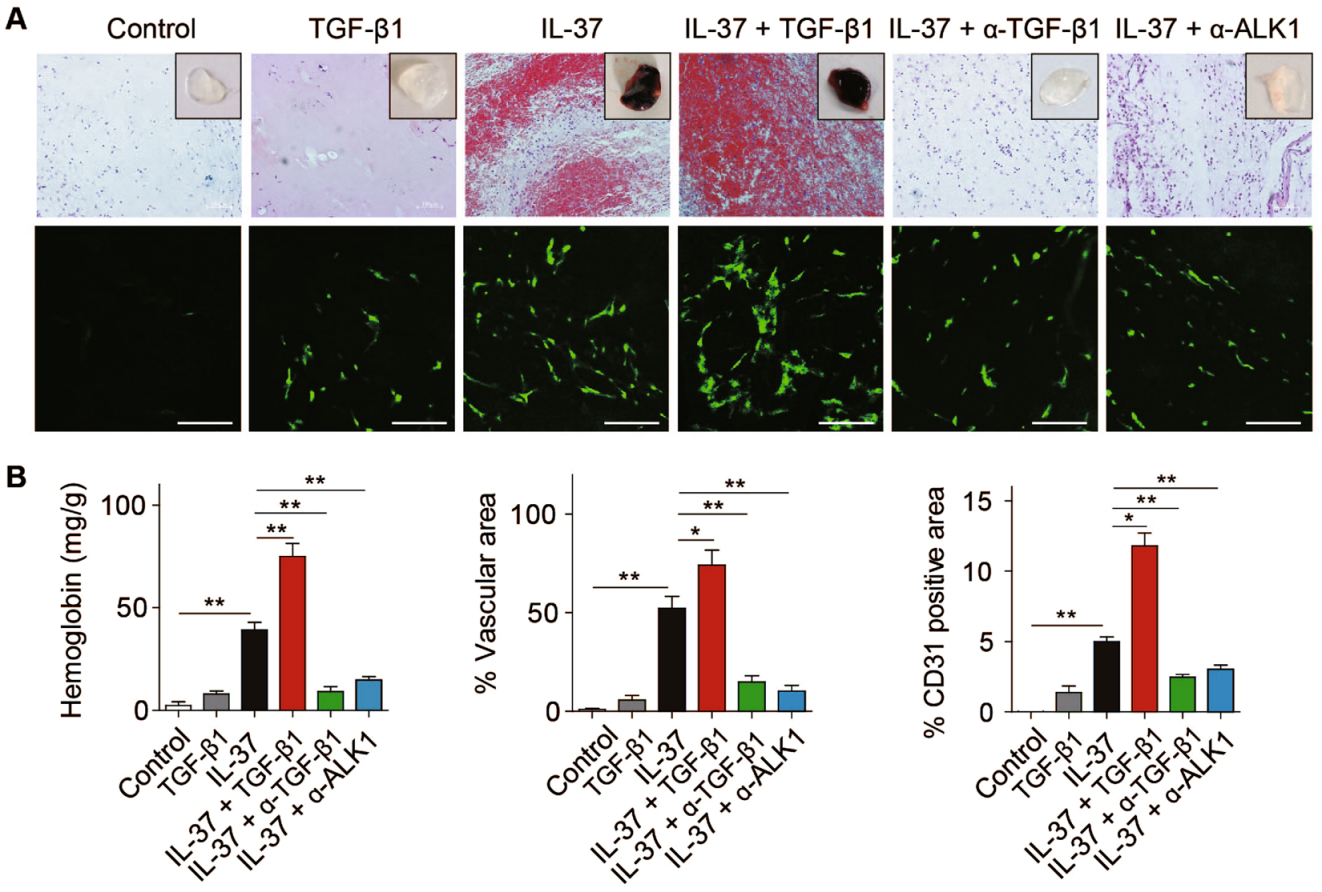
The pro-angiogenic effect of IL-37 was mediated by TGF-β signaling *in vivo*. (**A**) Representative images of Matrigel plugs incorporated with IL-37 (50 ng/mL) or/and TGF-β1 (50 ng/mL) or indicated neutralizing antibodies (10 μg/mL). Representative pictures of hematoxylin and eosin-stained sections and CD31 immunofluorescence-stained sections were shown. Scale bars, 100 μm. (**B**) Aggregate analysis of hemoglobin content, percentage of vascular area and percentage of CD31-positive area per field of Matrigel plugs. n = 8 per group. Data are presented as mean ± SEM. *P < 0.05, **P < 0.01 and ***P < 0.001.

We previously showed that IL-37 promoted angiogenesis in the mouse model of Matrigel plug and oxygen-induced retinopathy (OIR)^21^. To determine whether TGF-β augmented the pro-angiogenic effect of IL-37, combined incorporation of IL-37 and TGF-β1 in the Matrigel plug induced a substantial increase in vessel formation compared to IL-37-incorportated Matrigel plug (Fig. 5A and B). Furthermore, TGF-β-neutralizing antibodies and ALK1 inhibitor significantly blocked the angiogenic activity of IL-37 in Matrigel plug *in vivo* (Fig. 5A and B).

**Figure 6:**
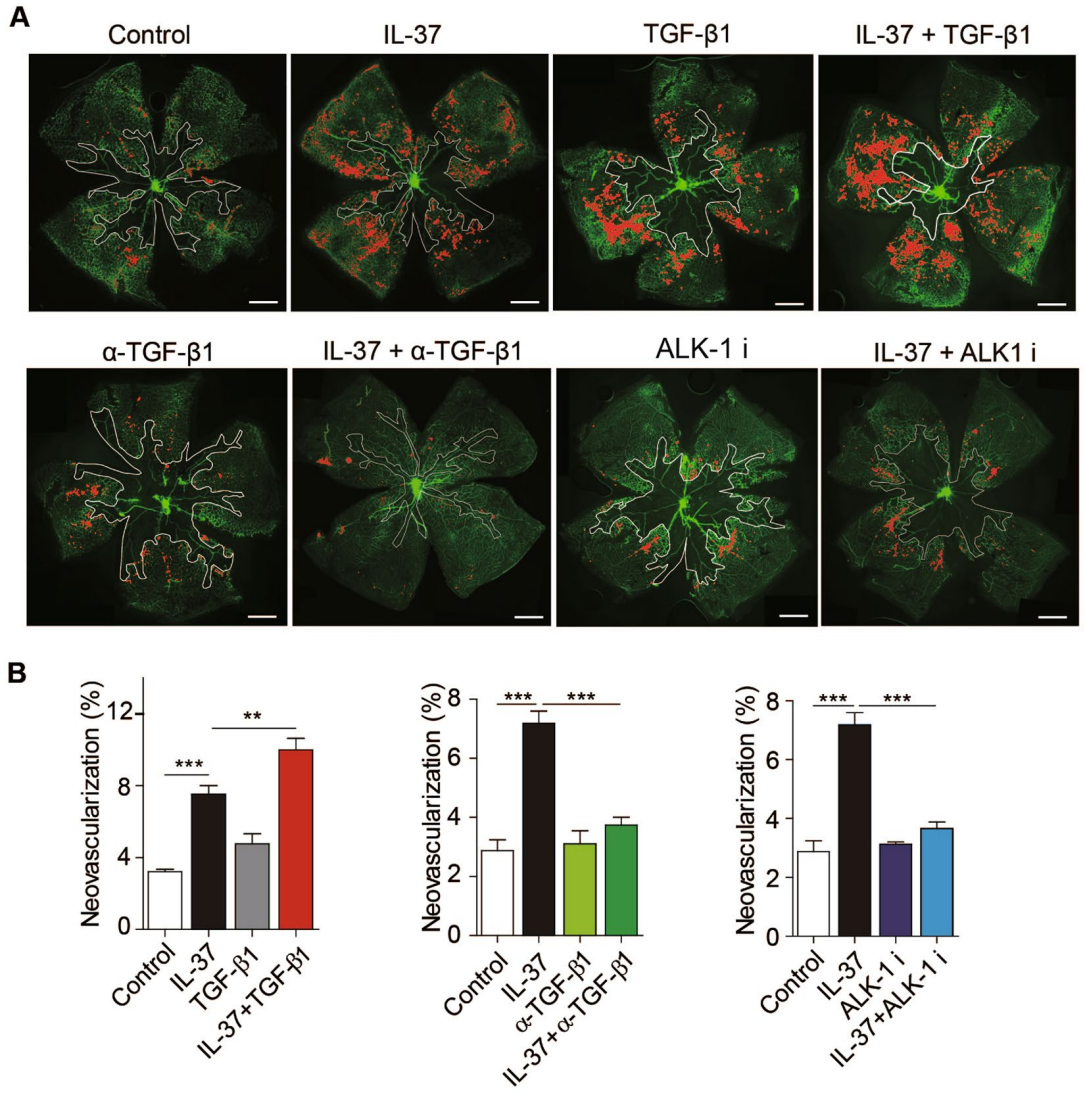
IL-37 promoted neovascularization through TGF-β signaling in OIR model. (**A**) Mice were administrated with 5 ng/g bodyweight IL-37 at postnatal day 12 (P12), P14 and P16 during normoxia phase. For blockade of TGF-β1 or ALK1, TGF-β1 neutralizing antibodies was administrated intraocularly at 0.5 μg per eye and ALK1 inhibitor LDN193189 was administrated intraperitoneally at 2 mg/kg bodyweight during normoxia phase. (n = 10 per group). (**B**) Disordered neovascular growth (tufts) highlighted in red in retina were quantified. Scale bars, 500 μm (lower magnification). Data are presented as mean ± SEM. *P < 0.05, **P < 0.01 and ***P < 0.001.

To further investigate the patho-physiological relevance of TGF-β-ALK1 signaling pathway in mediating the angiogenic effect of IL-37, TGF-β1 neutralizing antibodies were administrated into the vitreous of IL-37-treated mice in the OIR model during normoxia phase. We observed that TGF-β1-neutralizing antibodies significantly blocked the pro-angiogenic effect of IL-37 in OIR (Fig. 6A and B). As expected, intraperitoneal administration of ALK1 inhibitor also suppressed IL-37 induced upregulation of retinopathy (Fig. 6A and B). In addition, the requirement of TGF-β-ALK1 signaling pathway in IL-37-induced angiogenesis in mice retina development model were determined and similar results were observed (Fig. S10). Together, these results suggest that IL-37 promotes pathological angiogenesis through the TGF-β-ALK1 axis.

## Discussion

TGF-β plays a pivotal role in the regulation of vascular function. ALK1, the predominantly endothelial TGF-β type I receptor, plays a crucial role in vascular endothelial cells during angiogenesis. Activation of the ALK-1-Smad1/5/8 signaling induces expression of *ID1*, and subsequently activates pro-angiogenic signaling pathways in endothelial cells^2, 6^. IL-37 was reported to act as an important pro-angiogenic cytokine that promotes endothelial cell activation and pathological angiogenesis in our previous study. Extending our previous observation, we have identified a novel mechanism mediating the pro-angiogenic effect of IL-37. We establish that IL-37 induces pro-angiogenic responses, at least in part through TGF-β-ALK1 signaling pathway. TGF-β interacts with IL-37, and potently enhances the binding affinity of IL-37 to the ALK1 receptor complex, thus allowing IL-37 to signal through ALK1 to activate pro-angiogenic responses.

The data presented in this study support a hypothesis that TGF-β may act as the bridging molecule that mediates IL-37 binding to the ALK1 receptor complex. Using receptor-binding assay, we have demonstrated that in the presence of TGF-β, the binding of IL-37 to endothelial cells was remarkably enhanced, which is blocked by ALK1-specific antibodies. The interaction between IL-37 and TGF-β was further confirmed by pull-down assay and plate-bound assay. Moreover, TGF-β increased the affinity between IL-37 and the ALK1 receptor complex. IL-37 did not appear to interfere with the binding between TGF-β and the ALK1 receptor complex, supporting that IL-37 indirectly binds to ALK1 through interaction with TGF-β. We further examined the downstream signaling induced by ALK1 activation after IL-37 stimulation. IL-37 induced phosphorylation of Smad1/5/8 synergistically with TGF-β, as well as upregulation of targeted genes of Smad1/5/8. Of note, TGF-β has also been reported to induce activation of extracellular-regulated kinase (ERK) and phosphatidylinositol-3-kinase (PI3K)^22^. In line with this, we found that IL-37 activated Erk and Akt signaling in our previous study^21^. We further showed that TGF-β and ALK1 were required for IL-37-induced angiogenesis in functional analyses *in vitro* and *in vivo*. In addition, our results showed that IL-37 alone is able to induce pro-angiogenic response without TGF-β in ECs and in mouse model. The reason for this is probably due to the fact that TGF-β is contained in fetal bovine serum or secreted by cultured cells *in vitro* ^23^. And the concentration of TGF-β in mouse serum was around 2 ng/ml according to previous study^24^. Thus, even if TGF-β is not supplemented together with IL-37, trace amount of TGF-β may be sufficient to mediate the pro-angiogenic effect of IL-37. Taken together, these data show that IL-37 employs the TGF-β-ALK1 signaling to exert its pro-angiogenic effects.

To our knowledge, our study provides the first description of the requirement of TGF-β in the signaling of other cytokines. Our results showed that TGF-β significantly augmented IL-37-induced cell proliferation in a dose-dependent manner and blockade of TGF-β or ALK1 resulted in suppressed pro-angiogenic responses to IL-37, indicating the requirement of TGF-β for the pro-angiogenic effect of IL-37. Based on our previous results^21^, amounts as low as 10 pg/mL of IL-37 induced endothelial cell activation, and low concentration of IL-37 exhibit very potent activity *in vivo*, which is possibly due to the critical role of TGF-β in vascular function. Indeed, low concentrations of TGF-β induced EC activation, whereas high concentrations showed the opposite effects^25^. TGF-β was reported to act synergistically with other cytokines such as leptin, IL-13, TNF-α, epidermal growth factor (EGF), platelet-derived growth factor (PDGF)^26–30^. These synergistical effects were mediated through crosstalk between divergent signaling pathways activated by different receptors. Of note, a recent study reported that TGF-β signaling was modulated by an extracellular protein, LRG1, which modifies the formation of the TGF-β receptor complex, revealing the complexity of the regulation of TGF-β activity^31^. As the activity of TGF-β receptor complex is regulated by multiple mechanisms, the regulation of IL-37 signaling may involve participation of variable combinations of TGF-β receptors and coreceptors. Thus, precise molecular mechanism of interaction between IL-37 and TGF-β receptors remains to be established.

The regulatory effect of TGF-β-ALK1 signaling is highly complex and context- dependent. As an EC-restricted TGF-β type I receptor, although ALK1 has been recognized as a critic al regulator that induce pro-angiogenic signaling in endothelial cells, increasing evidence suggest the anti-angiogenic role of TGFβ-ALK1 in different contexts. For example, low concentration of TGF-β stimulates endothelial cell activation, whereas high concentration of TGF-β appears to suppress angiogenic response^32^. Blockade of ALK1 with adenovirus encoding ALK1-Fc resulted in increased neovascularization in developmental mouse retina^11^. This is in line with the study showing that inhibition of ALK-1 signaling by ALK-1-Fc or genetic deletion of *Alk1* significantly promotes neovascularization in OIR model^33^. However, our study showed that IL-37-induced angiogenesis is blocked by ALK1 inhibitor, suggesting that ALK1 plays an pro-angiogenic role in IL-37 induced angiogenesis. One explanation for this discrepancy is based on the fact that the inhibitory effect of ALK1 on angiogenesis is mediated by cooperation with other ligands or receptors. BMP9 and BMP10, other ligands for ALK1, was reported to inhibit bFGF or VEGF induced angiogenesis^34–36^. ALK1 was further reported to cooperate with Notch to inhibit angiogenesis^11, 12^. Notably, the inhibitory effect of ALK1 on angiogenesis is VEGF dependent, as combination of Alk1-Fc with antibodies neutralizing VEGF completely suppressed neovascularization^33^. These studies together suggest that ALK1 cooperates with other receptors and ligands to mediate negative feedback regulation on VEGF induced angiogenesis. Give that ALK1 exerts dual effects on angiogenesis, blockade of ALK1 with ALK-Fc or genetic depletion of ALK1 may abrogate interaction of ALK1 with other ligands or receptors, whereas ALK1 inhibitor that inhibits downstream signaling cascade of ALK1 may only suppress the pro-angiogenic effect of ALK1.

Our study has raised the intriguing possibility that IL-37 may be involved in many major biological processes regulated by TGF-β, such as cancer, fibrosis, tissue repair and inflammation. For example, studies suggest that recombinant IL-37 suppressed proinflammatory cytokine production in human PBMCs^13, 37^, which may involve the anti-inflammatory activity of TGF-β. Whether IL-37 regulates these biological processes thus remains to be further explored.

## Materials and Methods

### Reagents

Recombinant human IL-37 was purchased from Life Technologies (Thermo Fisher Scientific, Gaithersburg, MD) or R&D Systems (Minneapolis, MN). Possible TGF-β1 contamination in IL-37 was undetectable as determined by ELISA (R&D Systems, Minneapolis, MN). Anti-IL-37 antibodies were purchased from R&D Systems (Minneapolis, MN) or Abcam (Cambridge, MA). For Western blot analysis, antibodies against p-Smad1/5/8, Smad1/5/8, p-Smad2/3, Smad2/3 and β-actin were purchased from Cell Signaling Technology. Recombinant human TGF-β, BMP10 and GDF11 were purchased from Peprotech. Anti-human ALK1 antibodies, anti-human ALK5 antibodies, anti-mouse ALK1 antibodies, anti-mouse PECAM antibodies anti-human TGF-β and anti-mouse TGF-β antibodies were purchased from R&D Systems (Minneapolis, MN). ALK1 inhibitor LDN193189 was purchased from Sigma (St. Louis, MO). PE-conjugated streptavidin was purchased from Biolegend (San Diego, CA). Dylight549-conjugated streptavidin was purchased from Abcam (Cambridge, MA). Recombinant soluble chimeric protein containing the ectodomain of human ALK1 conjugated with IgG Fc (ALK1-Fc), chimeric protein containing the ectodomain of human ALK5 conjugated with IgG Fc (ALK5-Fc) and chimeric protein containing ectodomain of human TβRII conjugated with IgG Fc (TβRII-Fc) were purchased from Sinobiological (Beijing, China).

### Cells and culture

HUVECs were purchased from ScienceCell and maintained in ECM medium (ScienCell, Carlsbad, CA) according to company’s instructions. Specifically, ECM medium was supplemented with 5% FBS and endothelial cell growth supplement ((ECGS, Cat. No. 1052). Cells used for experiments were between passages 3 to 6.

### Enzyme-linked immunosorbent assay (ELISA)

HUVECs were treated with recombinant human 5 ng/ml TGF-β (Peprotech, Rocky Hill, NJ) or 5 ng/ml VEGF-A (R&D Systems, Minneapolis, MN) overnight. IL-37 protein in supernatant was determined with ELISA kit (R&D Systems, Minneapolis, MN) according to supplier’s instructions.

### Immunostaining

For Immunofluorescent staining of cultured HUVECs, cells were fixed, blocked with PBS supplemented with 5% BSA and incubated with 2 μg/ml α-IL-37 antibodies. Cells were then labeled with a fluorescein TRITC-conjugated secondary antibodies for 1 h at room temperature, rinsed in PBS and examined with fluorescent microscopy.

### Animals

C57BL/6 mice for oxygen-induced retinopathy model were purchased from Experimental Animal Centre of Sun Yat-sen University of Medical Science (Guangzhou, China). Care, use and treatment of all animals were in strict agreement with the guidelines of the Association for Research in Vision and Ophthalmology Statement for the Use of Animals in Ophthalmic and Visual Research and approved by the institutional animal care and use committee in Zhongshan Ophthalmic Center. For aortic ring experiments and retinal vasculature, C57BL/6 J mice were from Shanghai Laboratory Animal Center (SLAC, Shanghai, China). All experimental protocols were approved by the Animal Care and Use Committee of Tongji University.

### Western blot

For IL-37 expression, HUVECs were maintained in complete ECM without growth factors and then treated with 1 ng/ml or 5 ng/ml of TGF-β, BMP10 or GDF11 for 24 hours, and cell lysates were prepared using RIPA buffer and phosphatase and protease inhibitor cocktail (Roche, Mannheim, Germany). Proteins were separated using SDS-PAGE, transferred to nitrocellulose membranes and detected by antibodies against IL-37 (R&D Systems, Minneapolis, MN). For phosphorylation of Smad1/5/8 and Smad2/3, HUVECs were treated with 1 ng/ml of IL-37 in the presence or absence of 5 ng/ml of TGF-β1 for 10 minutes, 30 minutes and 60 minutes, and proteins were extracted and subjected to Western blot. Densitometric quantitation of was performed and analyzed using two-tailed Student’s *t* test.

### Proliferation assay

For the combined effect of TGF-β and IL-37 on HUVECs proliferation, 1 × 10^4^ HUVECs in 100 μl of ECM were seeded in 96 well plates at 1 × 10^4^/ml in serum-free ECM containing 0.1% BSA for overnight. The cells were then stimulated with combinations of different concentrations of IL-37 (0, 0.1, and 1 ng/ml) and TGF-β (0, 0.1, 1 and 10 ng/ml) for 48 hours. For the bioactivity of biotinylated IL-37, 1 × 10^4^ HUVECs in 100 μl of ECM were seeded in 96-well plates and maintained ECM containing 5% FBS. The cells were stimulated with different concentrations of biotinylated or unbiotinylated IL-37 or (0, 0.1, 0.5 1 and 5 ng/ml). For the effect of IL-37 on HUVECs proliferation, 1 × 10^4^ HUVECs in 100 μl of ECM were seeded in 96-well plates and maintained ECM containing 5% FBS without supplemented growth factors unless otherwise indicated. Antibodies neutralizing TGF-β (R&D Systems, Minneapolis, MN) or ALK1 (R&D Systems, Minneapolis, MN) were used at 10 μg/ml to block the effect of IL-37. Cell proliferation was determined by BrdU ELISA kit (Abcam, Cambridge, MA) according to company’s instructions. The statistical significance was determined by Student’s *t*-test.

### Matrigel tube formation assay

HUVECs were cultured in ECM containing 5% FBS without supplementary growth factors overnight. 1 × 10^4^ HUVECs in 100 μl of ECM were seeded on growth factor-reduced Matrigel (BD Biosciences, Bedford, MA) in 96-well plate. Media was supplemented with 1 ng/mL of IL-37 for 12 hours. For blockade experiments, TGF-β or ALK1 neutralizing antibodies were used at 10 μg/ml. ALK1 inhibitor LDN193189 was used at 0.5 μM. After 6 hours, the capillary-like structures were recorded with microscopy and tube length and branching points were measured with Photoshop.

### Aortic ring angiogenesis assay

Rings were sliced from aortae of 4-week old wild-type mice and sectioned into ~0.5mm pieces. Aortic rings were embedded in a 96-well plate containing collagen I gel (Millipore, Bedford, MA). The embedded rings were fed with M199 culture medium supplemented with 2.5% FBS. VEGF (5 ng/ml), IL-37 (1 ng/ml), anti-IL-37 antibodies (10 μg/ml), anti-ALK1 mAbs (10 μg/ml) and IgG (10 μg/ml) were added to the culture medium as indicated. Growth medium was changed every two days. The aortic ring sprouting was stained with anti-CD31 antibodies and the degree of sprouting was graded from 0 (least positive) to 5 (most positive) in a double-blinded manner.

### Matrigel plug assay

Sterile growth factor reduced Matrigel (BD Biosciences, Bedford, MA) supplemented with IL-37 was subcutaneously into the middle line of the back in mice. Antibodies against IL-37, TGF-β, ALK1 or IgG control (10 μg/ml) were mixed together with IL-37 in the Matrigel as indicated. For combined effect of IL-37 and TGF-β1, both IL-37 and TGF-β1 was used at 50 ng/ml. After 7 days, mice were anesthetized and Matrigel plugs were carefully pulled out after implantation and divided into 2 blocks. One Matrigel plug was weighed and digested with tissue homonizer and hemoglobin content was determined by Drabkin method (Sigma-Aldrich, St. Louis, MO). The other plug was fixed in 4% formalin and embedded in paraffin. Sections were stained with hematoxylin and eosin and then quantified under microscope. Vascular area was determined as the total lumen area of vessels, which are filled with erythrocytes. The percentage of vessel lumen area in 5 random fields per section was quantified. Immunofluorescent staining of sections was performed with anti-CD31 antibodies was performed to identify endothelial cells.

### Mouse model of OIR

Neonatal mice and their nursing mothers were placed in a 75% oxygen supply chamber from P7 to P12. Mice were returned to normal oxygen supply (21%) at P12. Mice were injected with PBS or IL-37 at 5 ng/gram bodyweight at P12, P14 and P16. For blockade of TGF-β1 or ALK1, TGF-β1 neutralizing antibodies was administrated intraocularly at 0.5 μg per eye at P12, P14 and P16. For blockade of ALK1, ALK1 inhibitor LDN193189 was administrated intraperitoneally at 2 mg/kg bodyweight at P12, P14 and P16. At P17, the whole retinal mounts were isolated and stained with Isolectin B4 (Sigma). Neovascular region (tufts) and avascular area were analyzed using ImageJ. Significant differences were determined using Student’s *t* test.

### Mouse retinal vascularization

Neonatal mice were administrated with IL-37 (1 ng/gram bodyweight) with or without TFG-β1 (1 ng/g bodyweight) at postnatal day 1 to day 4. For blockade of TGF-β1 or ALK1, TGF-β1 neutralizing antibodies was administrated intraocularly at 0.5 μg per eye and ALK1 inhibitor LDN193189 was administrated intraperitoneally at 2 mg/kg bodyweight. The whole retina mounts were isolated at P5 and stained with FITC-conjugated Isolectin B4. Retinal vascular area was analyzed with ImageJ.

### Protein biotinylation

The Biotin Protein Labeling Kit (R&D Systems, Minneapolis, MN) was used for biotinylation of IL-37 or TGF-β. 200 μg IL-37 or TGF-β was dissolved in 0.2 mL of biotin-7-NHS solution (20 mg/mL in PBS) and incubated for 2 hours at room temperature with gentle stirring. The reaction mix was then applied to Sephadex G-25 column. Labeled protein was then eluted with PBS and collected for experiments.

### Receptor binding assay

2.5 × 10^5^ cells (trypsinized HUVEC) were incubated with biot-IL-37, TGF-β, anti-ALK1 antibodies at indicated concentrations in binding buffer (PBS containing 1% bovine serum albumin and 0.1% NaN_3_) for 90 minutes at room temperature. Cells were washed with 200 μl ice-cold binding buffer, and incubated at 4 °C for 20 min with 1 μg/ml streptavidin (SAv)-PE in binding buffer protected from light. Cells were fixed in 2% paraformaldehyde (PFA) and bound biot-IL-37 or biot-TGF-β were quantified by flow cytometry.

### Immobilization binding assay

For detection of the binding affinity between IL-37 and TGF-β, ELISA plates (Nunc, Roskilde, Denmark) were coated with 2 μg/ml TGF-β1, anti-IL-37 capture antibodies or BSA (R&D Systems, Minneapolis, MN) diluted in PBS. Wells were blocked with 2% bovine serum albumin at 37 °C for 1 hr. Wells were then incubated with serial dilution of IL-37 at 4 °C overnight. Plates were washed and bound IL-37 was detected with streptavidin-HRP and peroxidase substrate. For detection of the affinity between ALK1 receptor complex and its ligands, recombinant ALK1 (5 μg/ml) and TβRII (5 μg/ml) proteins were incubated together and then coated to 96-well plates for overnight at 4 °C. Wells were washed and incubated with indicated concentrations of biot-IL-37 or biot-TGF-β. Bound biot-IL-37 or biot-TGF-β were detected with streptavidin-HRP and peroxidase substrate. Color reaction was determined at the absorbance at 405 nm.

### Co-immunoprecipitation

HUVECs were lysed in RIPA buffer and cell lysates were supplemented with IL-37 (50 nmol/l) and TGF-β (100 nmol/l), and then incubated with ALK1-specific antibodies-conjugated Dynabeads at 4 °C overnight with rotation. Immunoprecipitated proteins were extracted with SDS loading buffer and subjected to Western blot.

### Pull-down assay

Recombinant TGF-β1 (100 nmol/l) was mixed with recombinant IL-37 (50 nmol/l) in RIPA buffer and applied to anti-IL-37 antibodies-conjugated Dynabeads at room temperature for 20 minutes. Immunoprecipitated proteins were extracted with SDS loading buffer and subjected to Western blot. For detection of the interaction between IL-37 and the ALK1 or ALK-5 receptor complex, chimeric proteins of ALK1-Fc or ALK5-Fc and TGF-βRII-Fc were incubated to form receptor complex, which was then conjugated to protein A/G beads Dynabeads. The beads were then incubated with IL-37 (50 nmol/l) or/with TGF-β (100 nmol/l) for 2 hours at 4 °C with rotation. The beads were washed according to company’s instructions. Precipitated proteins were suspended in SDS sample buffer and subjected to Western blot analysis.

### ALK1 siRNA knockdown

For human ALK1 knockdown, siRNA oligonucleotides targeting ALK1 were purchased from Thermo Scientific (L-005302-00-0005) and control siRNA (D-001810-10-05) was used as a negative control for knockdown of ALK1 in HUVECs. For mouse ALK1 knockdown, siRNA targeting human ALK1 (Supplemental Table I) and scrambled siRNA was synthesized from Genepharm (Shanghai, China). Cells or aortic rings were transfected with 120 nmol/l scrambled siRNA or 120 nmol/l siALK1 comprised 40 nmol/l of the three antisense sequences using Lipofectamin RNAiMAX (Invitrogen, Carlsbad, CA) according to manufacturer’s instructions. After overnight recovery, aortic rings were incubated with 1 ng/ml IL-37 for 4 days, and vessel growth were recorded with microscope. The sprouting was scored from 0 (least positive) to 5 (most positive) in a double-blinded manner.

### mRNA isolation and qPCR

Cells were lysed with TRIzol (Sigma, St. Louis, MO), and mRNA was extracted and reverse transcribed according to manufacturer’s directions (Qiagen, Valencia, CA). Probes were listed in Supplemental Table II. Gene expression was normalized to β-actin.

### Data and statistical analyses

Data are presented as means ± SEM. Prism software was used for statistical analyses. Significant differences between paired samples were analyzed with the two-tailed Student’s *t* test. A *P* value of less than 0.05 was considered significant for all analyses.

## Electronic supplementary material

Supplementary Info
