# Supplementary material for: Interleukin 37 promotes angiogenesis through TGF-β signaling
Source: Sci Rep. 2017 Jul 21;7:6113. doi: 10.1038/s41598-017-06124-z (PMC5522482; doi:10.1038/s41598-017-06124-z)
Supplement: Supplementary file 1 — Supplementary Info [file 41598_2017_6124_MOESM1_ESM.pdf]

Interleukin 37 promotes angiogenesis through TGF-β signaling

One-sentence summaries: IL-37 promotes angiogenesis via TGF-β signaling

Mengmeng Zhao,[1]* Yongguang Hu,[1]* Jiayi Jin,[2] Ying Yu,[2] Shanshan Zhang,[1] Jingjing Cao,[1] Yuanfen Zhai,[1] Rongbin Wei,[1] Juanjuan Shou,[1] Wenping Cai,[1] Shangfeng Liu,[1] Xiaoping Yang,[3] Guo-Tong Xu,[1] Jianhua Yang,[1] David B. Corry,[4] Shao Bo Su,[1,2] Xialin Liu,[2 †] Tianshu Yang[1 †]

[1]Shanghai Tenth People's Hospital, Tongji University School of Medicine, Shanghai, 200092, China; [2]State Key Laboratory of Ophthalmology, Zhongshan Ophthalmic Center, Sun Yat-sen University, Guangzhou, China; [3]Johns Hopkins University School of Medicine, Baltimore, Unites States; [4]Department of Pathology and Immunology, Baylor College of Medicine, Houston, Unites States

*These authors contributed equally to this article.

[†]Corresponding author

Tianshu Yang, PhD, Associate Professor, Shanghai Tenth People's Hospital, Tongji University School of Medicine, Shanghai, China 200092. Tel: 86-21-65986790. Fax: 86-21-65986791. Email: tianshuy@tongji.edu.cn

or

Xialin Liu, MD PhD, Professor, State Key Laboratory of Ophthalmology, Zhongshan Ophthalmic Center, Sun Yat-sen University, Guangzhou, China 510060. Tel: 86-20-87330402. Fax: 86-20-87330403. E-mail: liuxl28@mail.sysu.edu.cn

SUPPLEMENTAL MATERIAL

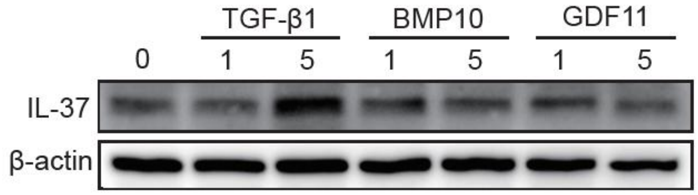

Figure S1. The effect of TGF-β family members on IL-37 expression. HUVECs were pre-starved under serum-free conditions without supplemented growth factors overnight and then treated with indicated concentrations of factors (TGF-β1, BMP10 and GDF11) for 24 hours. IL-37 expression was examined by Western blot. Blots are representative of three experimental replicates.

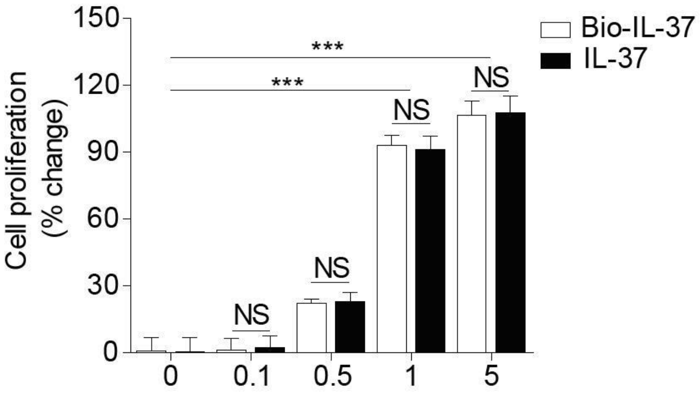

Figure S2. The bioactivity of biotinylated IL-37 and unbiotinylated IL-37. HUVECs were maintained in ECM containing 5% FBS without supplemented growth factors and stimulated with biotin-labeled or unlabeled IL-37 with indicated concentrations for 48 hours. Cell proliferation was determined by BrdU ELISA kit. Data are presented as mean ± SEM (n = 5 per group). *** P < 0.01. NS, not significant.

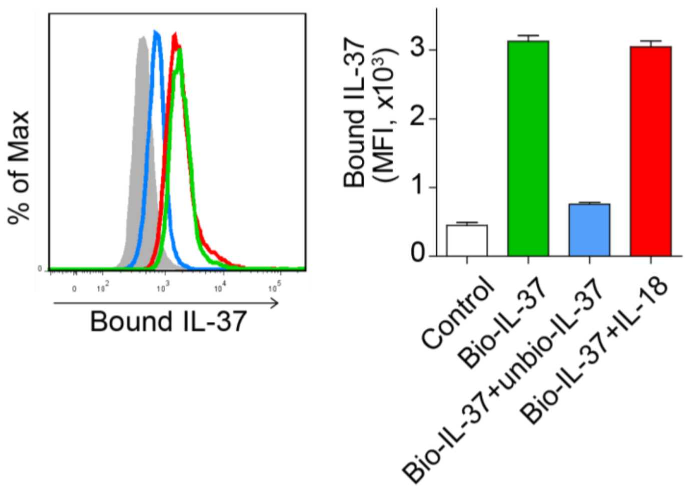

Figure S3. IL-18 did not compete the binding of IL-37. HUVECs were incubated with 1 μM bio-IL-37 in the presence of 5μM unbiotinylated IL-37 (blue histogram) or unbiotinylated IL-18 (red histogram). Bound biot-IL-37 was determined by flow cytometry using Streptavidin-PE (SAv-PE). Gray histograms indicated cells incubated with SAv-PE alone. The mean fluorescence intensity (MFI) was quantified (n = 4). *** $P < 0.01$; NS, not significant.

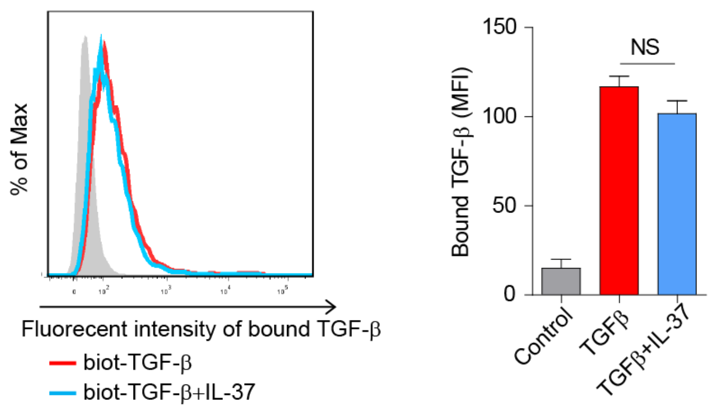

Figure S4. IL-37 does not affect the binding of TGF-β to HUVECs. IL-37 does not affect the binding of TGF-β to HUVECs. HUVECs were incubated with 1 μM biot-TGF-β in the presence (blue histogram) or absence (red histogram) of 1 μM unbiotinylated IL-37. Bound biot-TGF-β was determined by flow cytometry using SAv-PE. Gray histograms indicated cells incubated with SAv-PE alone. The mean fluorescence intensity (MFI) was quantified (n = 4). NS, not significant.

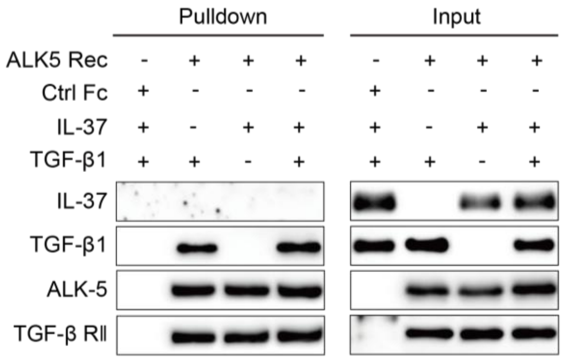

Figure S5. TGF-β does not affect the binding of IL-37 to ALK-5 receptor complex. The ALK5 receptor complex (pre-incubated ALK5-Fc and TGF-β RII-Fc) or control Fc were conjugated to protein A/G beads, which were then incubated with IL-37 in the presence or absence of TGF-β1. Immobilized proteins were resolved by Western Blot. Blots are representative of three experimental replicates.

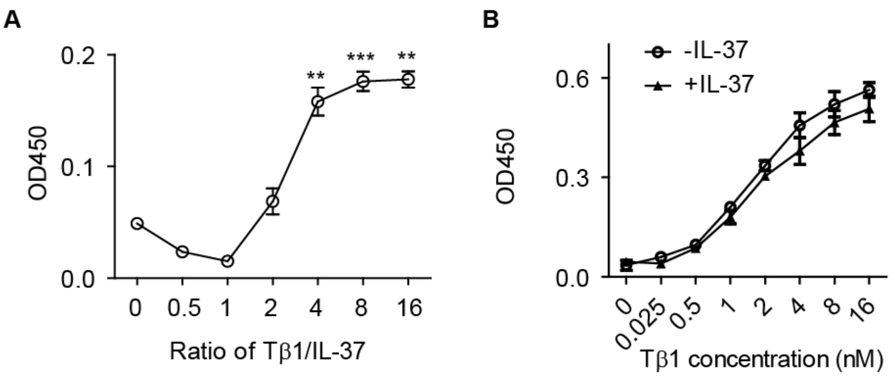

Figure S6. The binding affinity of TGF-β and IL-37 with the ALK1 receptor complex. (A) The 96-well ELISA plates were coated with the ALK1 receptor complex and then incubated with 5 nmol/l of bio-IL-37 in the presence of increasing concentrations of TGF-β (Tβ1). The binding of IL-37 was detected by Streptavidin-HRP. *n* = 4. (B) 96-well ELISA plates were coated with the ALK1 receptor complex and then incubated with increasing concentrations of biotinylated TGF-β (biot-Tβ1) in the presence or absence of 20 nmol/l of IL-37. The binding of biot-TGF-β1 was detected by Streptavidin-HRP. *n* = 4. Data were presented as mean ± SEM (*n* = 4 per group). ** *P* < 0.01; *** *P* < 0.001.

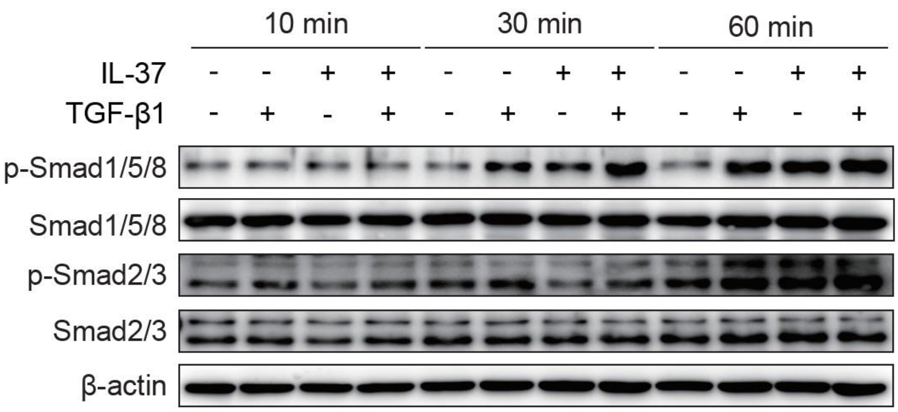

Figure S7. IL-37 and TGF-β1 synergistically stimulated phosphorylation of Smad1/5/8 rather than Smad2/3. HUVECs were pre-starved under serum-free conditions without supplemented growth factors overnight and then treated with IL-37 in the presence or absence of TGF-β1 for indicated time (10 min, 30 min and 60min). Phosphorylated Smad1/5/8 and Smad2/3 were determined by Western blot. Blots are representative of three experimental replicates.

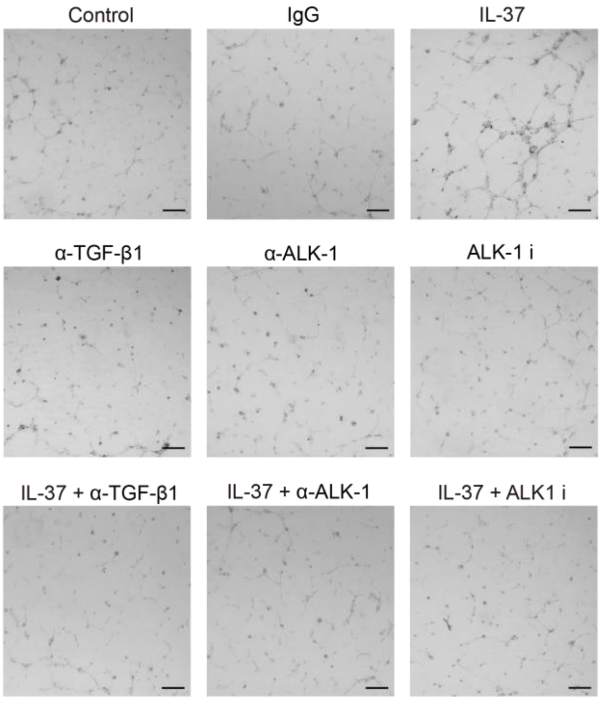

Figure S8. IL-37 promotes tube formation of HUVECs through TGF-ALK1 signaling. HUVECs were stimulated with IL-37 (1 ng/ml) in present or absent TGF-β1 antibody (10 μg/ml), ALK-1 antibody (10 μg/ml) and ALK-1 inhibitor (0.5 μM) for 12 hours. Representative images of tube structure were shown. Scale bars, 100 μm.

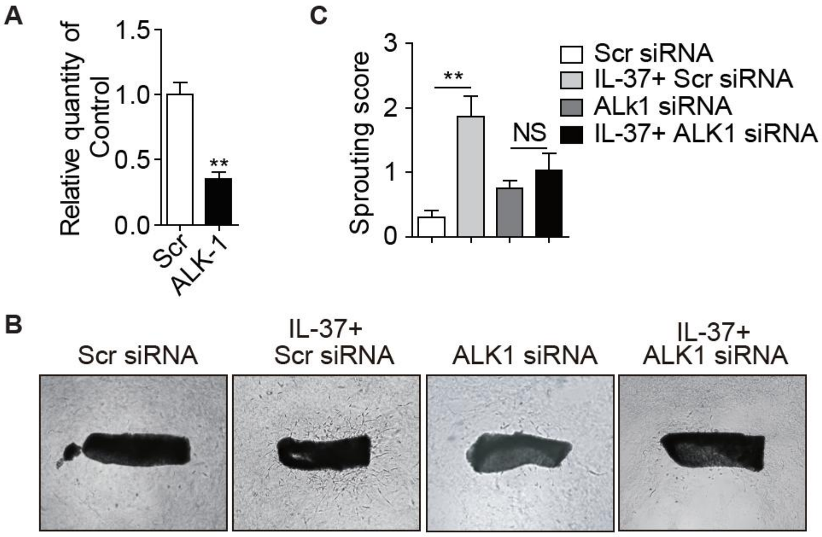

Figure S9. Knockdown of ALK1 reduced IL-37 (1 ng/mL) stimulated vessel growth from aortic rings. (A) Mouse aortic rings were transfected with 120 nmol/l scrambled siRNA or 120 nmol/l siALK1 composed of 40 nmol/l of the three antisense sequences using Lipofectamin RNAiMAX (Invitrogen) according to manufacturer's instructions. After overnight transfection, mRNA was isolated and *Alk1* mRNA level was quantified by quantitative PCR. Data were presented as mean ± SEM (n = 5 per group). $^{**}P< 0.01$. **(B)** Knockdown of ALK1 by siRNA inhibited IL-37-stimulated vessel growth from aortic rings. **(C)** Aggregate analysis of the sprouting. n = 10 per group. Data are presented as mean ± SEM. ** P < 0.01. NS, not significant.

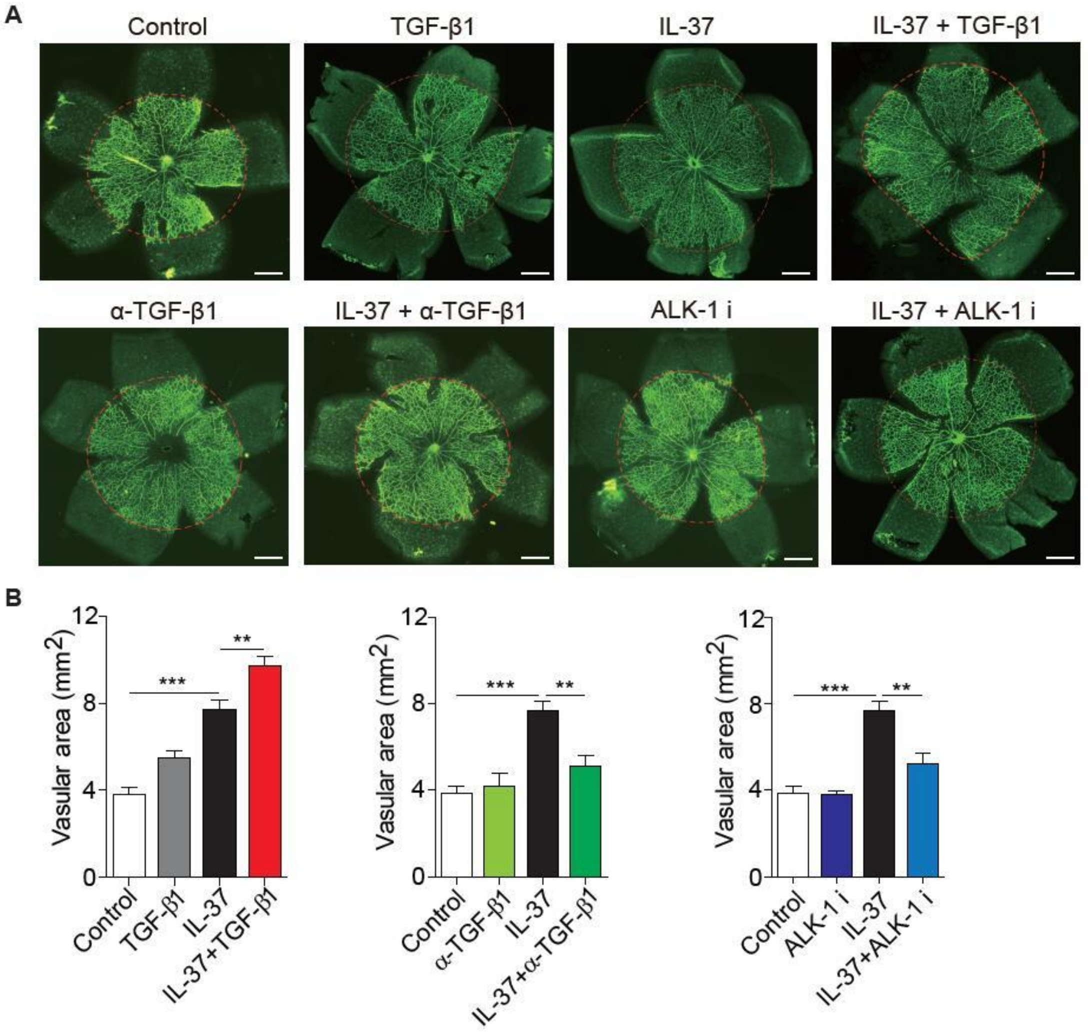

Figure S10. IL-37 promoted developmental angiogenesis through TGF-β signaling. (**A**) Neonatal mice were administrated with IL-37 (1 ng/g bodyweight) with or without TFG-β1 (1 ng/g bodyweight) from postnatal day 1 to day 4. For blockade of TGF-β1 or ALK1, TGF-β1 neutralizing antibodies was administrated intraocularly at 0.5 μg per eye and ALK1 inhibitor LDN193189 was administrated intraperitoneally at 2 mg/kg bodyweight. (n = 10 per group). (**B**) Vascular area of the retina whole mounts was assessed. Scale bars, 500 μm. Data are presented as mean ± SEM. * $P < 0.05$, ** $P < 0.01$ and *** $P < 0.001$.

Additional Tables

Table S1. siRNA sequences targeting ALK1

Primer name	Sense (5'-3')	antiense (3'-5')
ALK1_mouse_1	TGGTAGAGTGTGTGGGAAA	TTTCCCACACACTCTACCA
ALK1_mouse_2	CAGGAGAAGCAGCGGGATT	AATCCCGCTGCTTCTCCTG
ALK1_mouse_3	CCAGAGAAGCCCAAAGTGA	TCACTTTGGGCTTCTCTGG

Table S2. Primer sequences of real-time quantitative PCR

Primer name	Sequence (5' to 3')
hβ-actin_For	TTCCATATCGTCCCAGTTGGT
hβ-actin_Rev	CCAGGGCGTTATGGTAGGCA
mβ-actin For	GCTCGTTGCCAATAGTGATGACC
mβ-actin Rev	TGAGAGGGAAATCGTGCGTGAC
mALK1_For	GGCCTTTTGATGCTGTCG
mALK1_Rev	ATGACCCCTGGCAGAATG
hId1_For	TTCCTCTGGTTGACTGTTGTTCTTC
hId1_Rev	CTCTCTAAACTCCCTACGCCTTGTT
hId3_For	GGAGCTTTTGCCACTGACTCG
hId3_Rev	CTCCAGGAAGGGATTTGGTGAAGT
